# Probing antibody surface density and analyte antigen incubation time as dominant parameters influencing the antibody-antigen recognition events of a non-faradaic and diffusion-restricted electrochemical immunosensor

**DOI:** 10.1007/s00216-020-02417-x

**Published:** 2020-01-29

**Authors:** Jonathan Zorea, Rajendra P. Shukla, Moshe Elkabets, Hadar Ben-Yoav

**Affiliations:** 1 The Shraga Segal Department of Microbiology, Immunology and Genetics, Faculty of Health Sciences, Ben-Gurion University of the Negev, 8410501 Beer-Sheva, Israel; 2 Nanobioelectronics Laboratory (NBEL), Department of Biomedical Engineering and Ilse Katz Institute of Nanoscale Science and Technology, Ben-Gurion University of the Negev, 8410501 Beer-Sheva, Israel

**Keywords:** Antibody-antigen recognition events, Electrochemical impedance spectroscopy, Restricted diffusion, Immunosensors, Non-faradaic current, Capacitive detection

## Abstract

Electrochemical sensors based on antibody-antigen recognition events are commonly used for the rapid, label-free, and sensitive detection of various analytes. However, various parameters at the bioelectronic interface, i.e., before and after the probe (such as an antibody) assembly onto the electrode, have a dominant influence on the underlying detection performance of analytes (such as an antigen). In this work, we thoroughly investigate the dependence of the bioelectronic interface characteristics on parameters that have not been investigated in depth: the antibody density on the electrode’s surface and the antigen incubation time. For this important aim, we utilized the sensitive non-faradaic electrochemical impedance spectroscopy method. We showed that as the incubation time of the antigen-containing drop solution increased, a decrease was observed in both the solution resistance and the diffusional resistance with reflecting boundary elements, as well as the capacitive magnitude of a constant phase element, which decreased at a rate of 160 ± 30 kΩ/min, 800 ± 100 mΩ/min, and 520 ± 80 pF × s^(α-1)^/min, respectively. Using atomic force microscopy, we also showed that high antibody density led to thicker electrode coating than low antibody density, with root-mean-square roughness values of 2.2 ± 0.2 nm versus 1.28 ± 0.04 nm, respectively. Furthermore, we showed that as the antigen accumulated onto the electrode, the solution resistance increased for high antibody density and decreased for low antibody density. Finally, the antigen detection performance test yielded a better limit of detection for low antibody density than for high antibody density (0.26 μM vs 2.2 μM). Overall, we show here the importance of these two factors and how changing one parameter can drastically affect the desired outcome.

**Electronic supplementary material:**

The online version of this article (10.1007/s00216-020-02417-x) contains supplementary material, which is available to authorized users.

**Figure:**
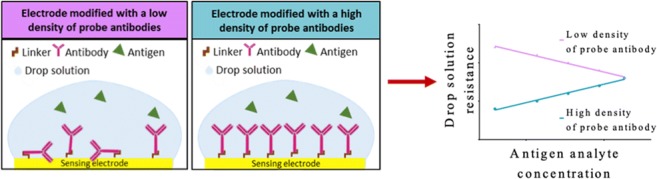
Graphical abstract

## Introduction

Electrochemical sensors based on antibody-antigen recognition events are commonly used for identifying many biological markers [1–3]. These sensors utilize various detection mechanisms that are classified based on the output electrical signal: current (namely, amperometric detection), potential (namely, voltammetric detection), and impedance (namely, conductometric detection) [4–6]. Among these three detection mechanisms, conductometric detection is known for its high sensitivity to physicochemical reactions at the bioelectronic interface and the ability to differentiate these reactions by removing background effects [7].

Antibody-conjugated electrochemical biosensors (‘immunosensors’) are used in many fields, including environmental protection, biotechnology, drug screening, food safety, security, veterinary medicine, and the monitoring and diagnosis of diseases [8]. Moreover, these biosensors are considered to have several advantages such as short test times and low test costs [9]. These advantages are achieved by covalently binding the antibody probe to the electrode surface, and hence reducing the amount of antibody needed for the test and increasing its accessibility [10]. Moreover, a unique class of these immunosensors does not require adding electro-active species to observe antibody binding events, namely, non-faradaic detection, enabling true label-free detection. Despite the improved detection performance of non-faradaic electrochemical immunosensors, it has been reported that changing one parameter of the antibody-electrode (‘bioelectronic’) interface can drastically affect the detection performance; therefore, this should be carefully studied. Although some electrochemical parameters of the bioelectronic interface, such as the electrode surface area [11], the electrode material [12], or the linker concentration [13], have already been reported to drastically affect the resulting sensitivity, only limited information has been reported on biological components, such as the antibody probe density and the antigen analyte incubation time.

Here, we investigated how the antibody-antigen recognition events in a non-faradaic electrochemical immunosensor depend on the antibody probe density at the electrode’s surface and on the antigen analyte incubation time. To address this aim, we used high and low antibody concentrations and six different incubation times. To analyze the measured results, we used a restricted diffusion-based electrical equivalent model [14], since we found that it better describes the system, and harnesses the influence of the microliter-volume solution (a ‘drop solution’) on the molecular diffusion. We showed that as the incubation time of the antigen increased, both the solution and diffusional resistance elements and the capacitive magnitude of a constant phase element decreased at a rate of 160 ± 30 kΩ/min, 800 ± 100 mΩ/min, and 520 ± 80 pF × s^(α-1)^/min, respectively. Using atomic force microscopy (AFM), we also showed that the antibody concentration affects the electrode’s roughness: a high antibody concentration yielded a root-mean-square (RMS) roughness value of 2.2 ± 0.2 nm, and a low antibody concentration resulted in a value of 1.28 ± 0.04 nm. Furthermore, we showed that as we increased the antigen concentration, the solution resistance element increased for the high antibody concentration and decreased for the low antibody concentration. Finally, the antigen detection performance test yielded a better limit of detection (LOD) for low antibody density than high antibody density (0.26 μM vs 2.2 μM). Overall, we showed the importance of these two factors and how changing one parameter can drastically affect the desired outcome.

## Materials and methods

### Chemicals and materials

Dithiobis (succinimidyl) propionate (DSP) was purchased from TCI (D2473) and dissolved in sterile dimethyl sulfoxide (DMSO, Sigma D8418). Potassium hexacyanoferrate(II) trihydrate (‘ferrocyanide’) and potassium hexacyanoferrate(III) (‘ferricyanide’) were purchased from Merck (P3289, 244,023). We used rabbit anti-human IL-2 antibody (500-P22, Peprotech) as the primary antibody and Alexa Fluor® 488 AffiniPure Goat Anti-Rabbit IgG (Jackson ImmunoResearch, 111-545-144) as the secondary antibody. Recombinant human IL-2 (200-02, Peprotech) was used as the antigen in this study. Sterile phosphate-buffered saline (PBS) was purchased from Biological Industries (02-023-1A). Tris-HCl buffer was made by dissolving TRIS (hydroxymethyl aminomethane, Bio-Lab 20,092,391) with double-distilled water (DDW, with resistivity of 18.3 MΩ × cm). Thin-film multi-single electrodes (ED-SE-8x-Au) and the multi-electrode chip platform (ED-ME-CELL) were purchased from Micrux™ and were used in all the experiments. The electrode chips included gold working and counter electrodes, printed on the thin-film chip. All experiments were performed using a VSP-300 potentiostat (BioLogic, Ltd.), and were referenced to the RE-1B reference electrode (Ag/AgCl) (3 M NaCl, ALS Co., Ltd.).

### Antibody complex assembly onto an electrode

**Fig. 1:**
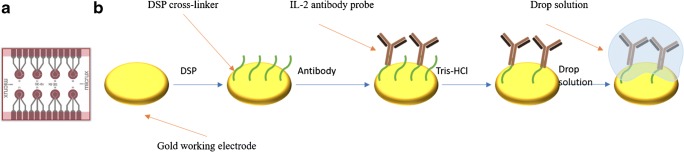
Sensing antibody complex assembly. (**a**) The on-demand Micrux™ chip used for the tests. (**b**) Illustration of the assembly process. DSP linker (green wavy lines) was allowed to bind onto the gold surface (gold disc shape) for 30 min. Then, the antibody (brown ‘Y’ shape) was incubated on the electrode for 2 h. Finally, the electrode was washed with Tris-HCl to remove any excess of free N-Hydroxysuccinimide (NHS) groups

We used the Micrux™ multi-electrode chip platform and an on-demand thin film, eight-single gold electrode chip. The electrodes are arranged in a 2 × 4 pattern, and the surface area of each working electrode was 0.8 mm^2^ (Fig. 1a). We chose to immobilize the IL-2 antibody to the gold electrode surface using the DSP cross-linker molecule. DSP contains a cleavable disulfide bridge in its spacer arm. One side of it interacts with the gold surface, forming a strong thiol, and the other interacts with the primary amine residues of the antibody, due to its NHS-ester reactive ends, forming a stable amide linkage. Following a 30-min incubation with a 10 mM solution of DSP dissolved in 100% DMSO, we allowed the antibody to form a stable bond to the electrode for another 2 h at room temperature. Next, we rinsed the electrode surface with a 10 mM pH 8.0 Tris-HCl buffer to remove any cross-linker byproducts (NHS leaving groups). Lastly, we dropped 5 μl of the antigen solution and performed the desired measurement (the whole procedure is also described in Fig. 1b).

### Optical characterization of the antibody-modified electrode

We incubated the antibody-conjugated electrode (an electrode that was not conjugated with antibodies was used for the control) with a secondary antibody, conjugated with an Alexa 488 fluorophore for 1 h at room temperature. Next, we observed the chip under a Zeiss Axio Observer 7 fluorescence microscope by exposing the chip to the bright light of the background image and then we excited the sample with a green LED and detected the secondary antibody using a filter suitable for 519-nm emission.

### Electrochemical characterization of the antibody-modified electrode

Cyclic voltammetry (CV) measurements were repeatedly used in a potential range of 0.23–0.48 V vs. Ag/AgCl at room temperature with a scan rate of 100 mV/s. Differential pulse voltammetry (DPV) measurements were performed by sweeping the potential between 0 and 0.5 V vs. Ag/AgCl with a 50-mV pulse amplitude, 9-ms pulse width, 5-mV step height, and a 100-ms step time. Electrochemical impedance spectroscopy (EIS) measurements were carried out with a fixed potential of 0.23 V vs. the Ag/AgCl reference electrode, at a frequency range of 1 MHz to 1 Hz with a 25-mV amplitude and three measurement points per decade. To characterize the bioelectronic interface during the antibody assembly procedure, CV, EIS, and DPV measurements were recorded using a 5 mM ferrocyanide-ferricyanide solution dissolved in PBS. After each assembly step, the chip was placed in the platform, 5 μl of ferrocyanide-ferricyanide solution was dropped on the working and the counter electrodes, the reference electrode was placed inside the solution above the working electrode, and then the measurement was performed.

### Atomic force microscopy measurements of the modified electrodes

We used the MFP-3D-BIO Atomic Force Microscope system (Asylum Research) together with HQ-300-Au (Asylum Research) and AC240TS (Olympus) probes. In addition, we used the AC-mode in ambient conditions (partly in repulsive mode, ‘tapping mode’, and partly in attractive mode) and scanned an area of 1 μm^2^.

### Antibody incubation characterization and antigen detection measurements using non-faradaic electrochemical impedance spectroscopy

We chose two antibody concentrations, 1 μg/μl (‘high density’) and 100 pg/μl (‘low density’), and incubated each with five sequential concentrations of the antigen (0, 50 fg/μl, 5 pg/μl, 500 pg/μl, and 20 ng/μl). A 5-μl drop of each concentration of the antigen was incubated for 3 min, and then a single EIS measurement was recorded (with a fixed potential of 0.23 V vs. the Ag/AgCl reference electrode and at a frequency range of 1 MHz to 1 Hz, at a 25-mV amplitude, and three measurement points per decade).

## Results and discussion

### Antibody complex is assembled onto an electrode

**Fig. 2:**
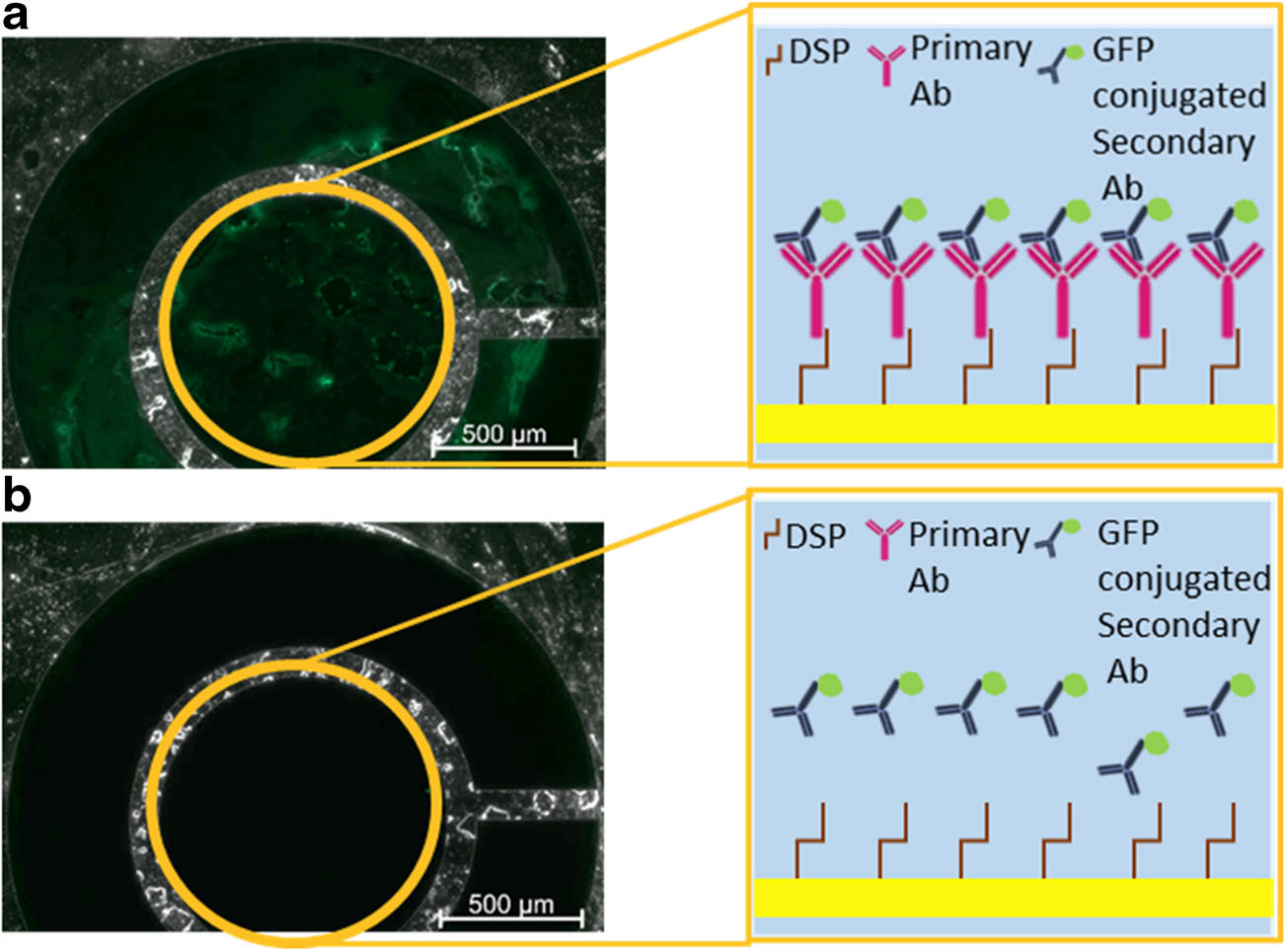
Optical characterization of the antibody-modified electrode. Optical images of electrodes incubated with a secondary GFP-conjugated antibody following an incubation with DSP and the primary antibody (**a**) or only with DSP (**b**). In the illustration, the electrode is denoted as a yellow square, DSP is denoted as a brown stick, the primary antibody as a pink ‘Y’ shape, and the secondary antibody as a dark blue ‘Y’ shape with a small green circle on its base

In our model we used the well-described [15–17] cross-linker molecule DSP to conjugate the probe antibody to the electrode surface. We characterized the assembly of the antibody onto the working electrode using optical and electrochemical methods. To first validate the conjugation of the antibody to the chip, we incubated the antibody-conjugated electrochemical chip with an Alexa 488 secondary antibody. To avoid a background signal due to nonspecific binding of the secondary antibody, we performed a control experiment by incubating an electrode modified only with DSP, with the secondary antibody. Imaging both of the electrodes exhibited green fluorescence only for the electrode modified with the primary antibody (Fig. 2a) and not for the control electrode (Fig. 2b). The obtained results validated the successful conjugation and functional stability of the antibody structure.

**Fig. 3:**
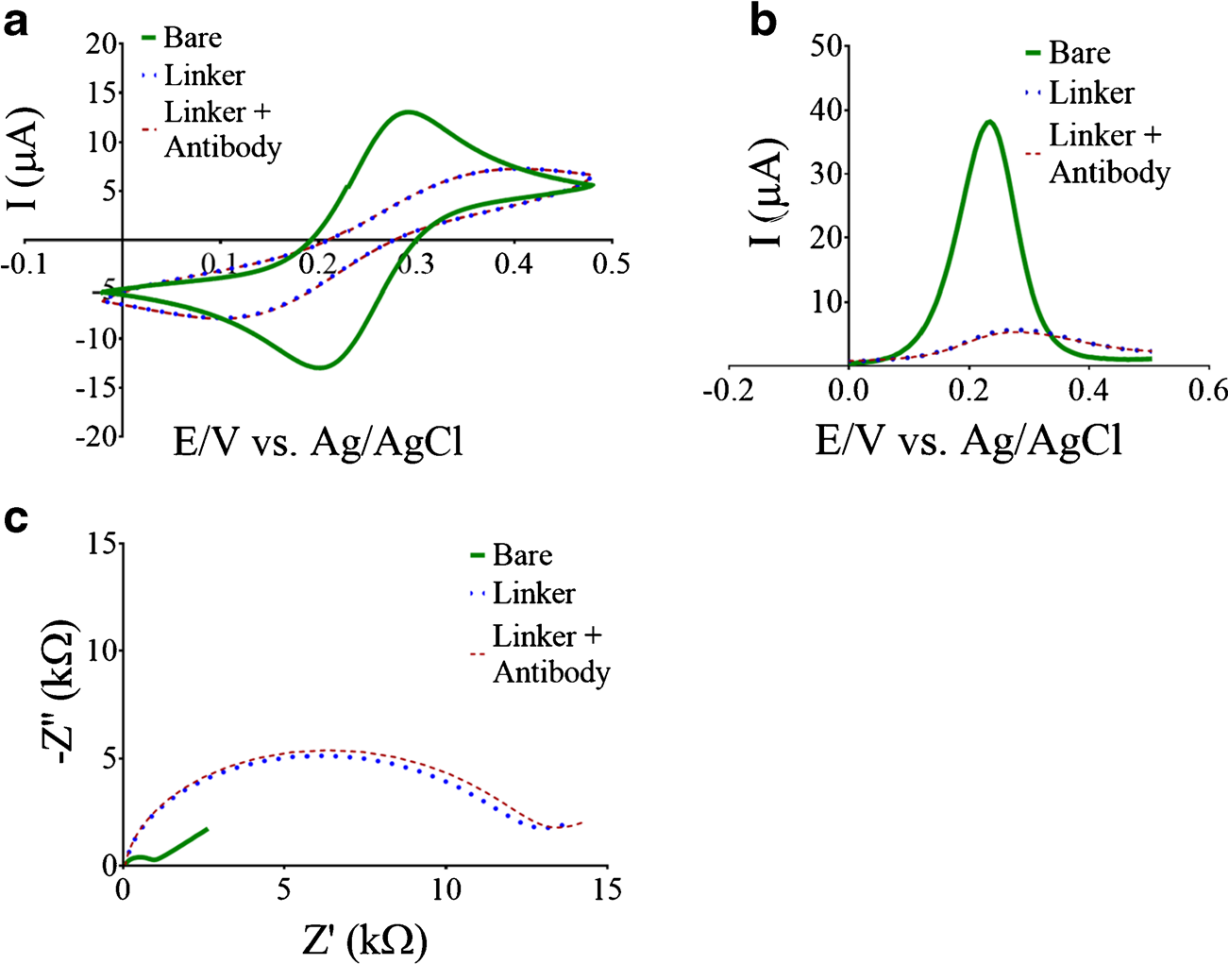
Electrochemical characterization of the antibody-modified electrode. Cyclic voltammograms (**a**), differential pulse voltammograms (**b**), and electrochemical impedance spectrograms (**c**) recorded for a bare electrode (solid green) and electrodes modified with either 10 mM DSP linker (dotted blue) or 10 mM DSP linker +1 μg antibody complex (dashed brown). Measurements were recorded by dripping 5 μl of a 5 mM ferrocyanide-ferricyanide solution dissolved in PBS on the working and the counter electrodes. The reference electrode was placed inside the solution above the working electrode prior to performing the measurement. Cyclic voltammograms were recorded at a potential range of 0.23 to 0.48 V vs. Ag/AgCl, at room temperature, and a scan rate of 100 mV/s. Differential pulse voltammograms were recorded at potential sweeps between 0 and 0.5 V vs. Ag/AgCl, at a 50 mV pulse amplitude, a 9-ms pulse width, a 5-mV step height, and a 100-ms step time. Electrochemical impedance spectrograms were recorded at a fixed potential of 0.23 V vs. the Ag/AgCl, at a frequency range of 1 MHz to 1 Hz, 25-mV amplitude, and three measurement points per decade

We characterized the assembly of the antibody onto the electrode using three complementary electrochemical techniques—CV, DPV, and EIS—and in the presence of the commonly used ferrocyanide/ferricyanide redox couple. For each technique, measurements were recorded after each step of the assembly process (i.e., a bare electrode, a DSP-conjugated electrode, and a primary antibody-DSP-conjugated electrode). In the recorded cyclic voltammograms (Fig. 3a), we observed a decrease in the cathodic and anodic peak current values after DSP conjugation, with no additive change after the antibody conjugation. Moreover, we observed a trend similar to the cyclic voltammograms in the recorded differential pulse voltammograms (Fig. 3b). Nyquist plot representation of the recorded electrochemical impedance spectrograms (Fig. 3c) showed a semi-circle that had a bigger radius after DSP conjugation, with a slightly additive increase after the antibody conjugation. By using the increased observed radius and the known equivalent electrical circuit of Randles cell [18], we showed that the charge transfer resistance increases for each modification step [19]. Therefore, these results led us to use EIS for our additional measurements, since they could better discriminate between each step.

### Antigen incubation time affects the bioelectronic interface

**Fig. 4:**
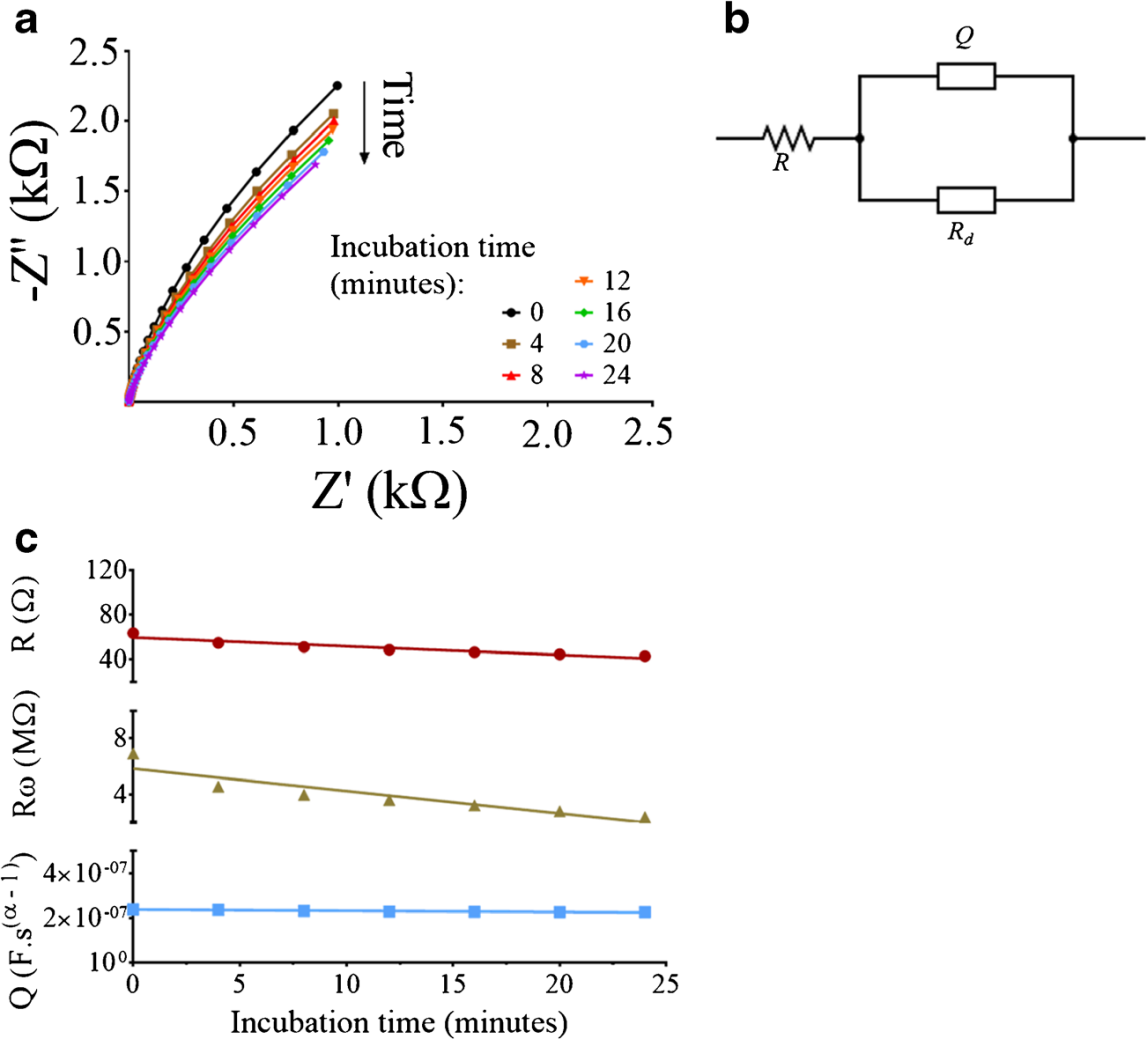
Antigen incubation time affects the bioelectronic interface. (**a**) EIS was measured six times, once every 4 min for 24 min total. Concentrations of 1 μg antibody and 50 fg antigen were used for these measurements. The measurements were performed at a fixed potential of 0.23 V vs. the Ag/AgCl, at a frequency range of 1 MHz to 1 Hz, a 25 mV amplitude, and three measurement points per decade. (**b**) The equivalent electrical circuit used to fit the physicochemical bioelectronic interface. *R* is the resistance element, *Q* is the constant phase element, and *R*_*d*_ is the anomalous diffusion Ia element with a reflecting boundary. (**c**) The dependence of *R*_*휔*_ (yellow), *R* (red), and *Q* (blue) on the incubation time. Fitting to the equivalent electrical circuit was done using the Z-Fit Bio-Logic tool. Linear regressions and data processing were performed using Prism Graphpad 7.0

We evaluated the effect of the antigen incubation time on the electrochemical system using non-faradaic EIS. Briefly, we incubated the high-density antibody-modified electrode with a fixed concentration of the antigen and measured the resulting electrochemical signal every 4 min, which is a total of 24 min (Fig. 4a). The impedance measured at lower frequencies revealed an appreciable difference between the measurements. We could not see this difference at high frequencies. Moreover, the impedance at low frequencies decreased for longer incubation durations. We analyzed the recorded EIS measurements with an equivalent electrical circuit (EEC) method describing the physicochemical interface at the electrode-antibody interface (Fig. 4b). The EEC consisted of a constant phase element (*‘Q’*) representing the double layer capacitance (*Q* = *1/Y(iω)*^*α*^, where *Y* is the capacitive magnitude, *ω* is the radial frequency, and *α* is the capacitive exponent index), a resistance element (*‘R’*) representing the solution resistance, and an anomalous diffusion Ia element with reflecting boundary (*‘R*_*d*_*’*) [14] represented by Eq. 1:

1 $$ {R}_d(s)=R{\left({\omega}_d/s\right)}^{\gamma /2}\coth \left[{\left(s/{\omega}_d\right)}^{\gamma /2}\right], $$

where *R*_*d*_ is the impedance component of the anomalous diffusion Ia element with reflecting boundary, *R*_*ω*_ = *L/qAD (dE/dc)*_*0*_, where *L* is the diffusion layer thickness, *q* is the charge that crosses the interface layer per diffusing particle, *A* is the electrode area, *D* is the diffusion coefficient, *(dE/dc)*_*0*_ is the diffusion overvoltage per concentration of diffusing species following local equilibrium conditions, *ω*_*d*_ is the characteristic frequency, *γ* is the derivation index, and *s = iω* and *ω = 2πf,* and where *f* is the frequency. We used an anomalous diffusion method to model the bioelectronic interface, since we assumed that the concentration of the antigen in the droplet is not maintained and changes continuously due to antibody-antigen binding reactions at the electrode surface. Moreover, a reflecting boundary was used to include the effect of the droplet’s boundary at the solution-air interface that becomes dominant at long measurement durations, and its values are of the same magnitude as the diffusion length (proportional to (*Dt*)^1/2^, where *t* is the duration of the measurement) [19, 20]. The calculated EEC components showed a negative relationship with the incubation time (Fig. 4c). The resistive component of *R*_*d*_ (i.e., *R*_*ω*_) was negatively and linearly related to the incubation time (slope: −160 ± 30 [kΩ/min] and Y-intercept: 5.85 ± 0.45 [MΩ], *R*^2^ = 0.84). Moreover, both *R* and the capacitive magnitude of *Q* (i.e., *Y*) were negatively and linearly related to the incubation time (slope: −800 ± 100 [mΩ/min] and Y-intercept: 59.5 ± 1.7 [Ω], *R*^2^ = 0.90; slope: −520 ± 80 [pF × s^(α-1)^/min] and Y-intercept: 234 ± 1 [nF × s^(α-1)^], *R*^2^ = 0.89, respectively). Other components of the EEC did not show a significant dependence on the incubation duration (see Fig. S1 in the Electronic Supplementary Material, ESM). The decreasing *R*_*휔*_ and *R* values for longer incubation durations can be due to the increasing concentrations of the electrolyte and the antigen resulting from the decreasing volume of the drying droplet. The decreasing *Y* values for longer incubation durations can be due to the increasing density of the positively charged [21, 22] antigen binding events in relation to the antibodies.

### Antibody density on the electrode affects the antigen detection performance

**Fig. 5:**
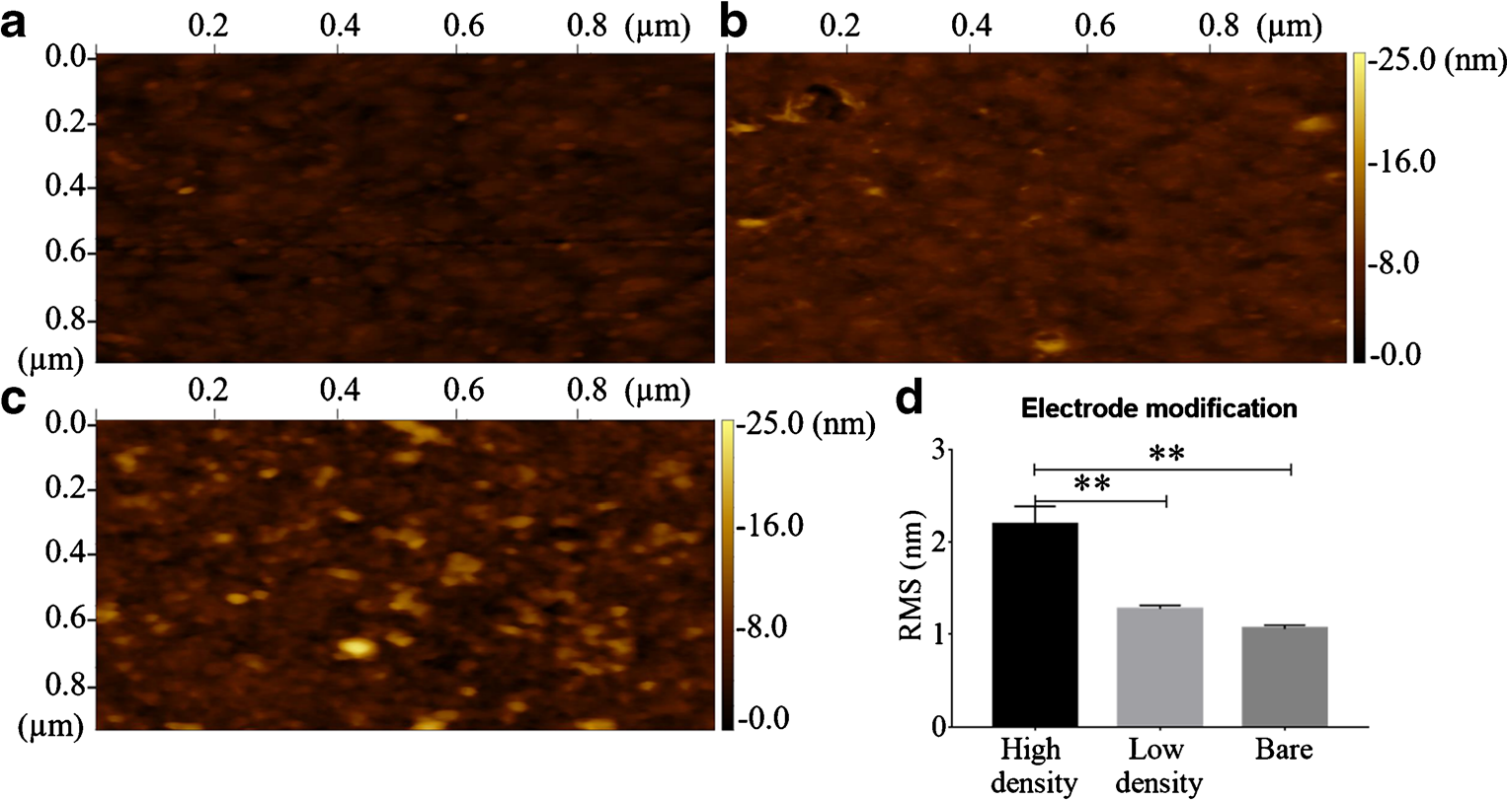
Antibody concentration affects the roughness of a modified gold electrode. Force micrographs of a bare electrode (**a**) and electrodes modified with low (**b**) and high (**c**) concentrations of antibody (100 pg and 1 μg, respectively). An area of 1 μm^2^ was scanned each time. (**d**) Ordinary one-way analysis of variance (ANOVA) of the surface roughness (RMS), differentiating between each modification. Three separate locations on the electrode for each condition were used for the analysis; they are represented as the mean with SD. Adjusted *p*-values of 0.05 (*), 0.01 (**), 0.001 (***), and 0.0001 (****) were considered statistically significant

The antibody probe’s density on the surface of the electrode can affect the probe’s conformation and the measured electrochemical signal. For example, a low density of single-stranded DNA probes collapses on the surface of the electrode into a horizontal conformation, whereas high density results in a vertical standing conformation [20]. We hypothesized that this conformation trend affects the way the antibodies assemble on the electrode during the conjugation steps. Therefore, we first characterized the structural morphology of the electrode modified with high and low densities of antibodies using AFM scans. Next, we compared the RMS roughness of a bare electrode (Fig. 5a) with low (Fig. 5b) and high (Fig. 5c) assembly densities of antibodies. We discovered that the high-density antibody-modified electrode resulted in increased RMS (2.2 ± 0.2 nm) than the low-density antibody-modified electrode (1.28 ± 0.04 nm). The RMS measured under the high-density condition was even higher than that of the bare electrode (1.06 ± 0.04 nm) (Fig. 5d). When we measuring the average height of the events for each of the three conditions, we noticed the same trend, but it was not significant (ESM Fig. S2). Taken together, our results indicate that antibodies under low-density conditions are arranged differently on the electrode than under high-density conditions. According to a review published by Trilling et al. [23], one possible explanation for these changes is the antibody’s orientation. We suspect that the low-density antibodies are found in a horizontal conformation, whereas the high-density conditions may result in antibodies that assemble in a vertical conformation.

**Fig. 6:**
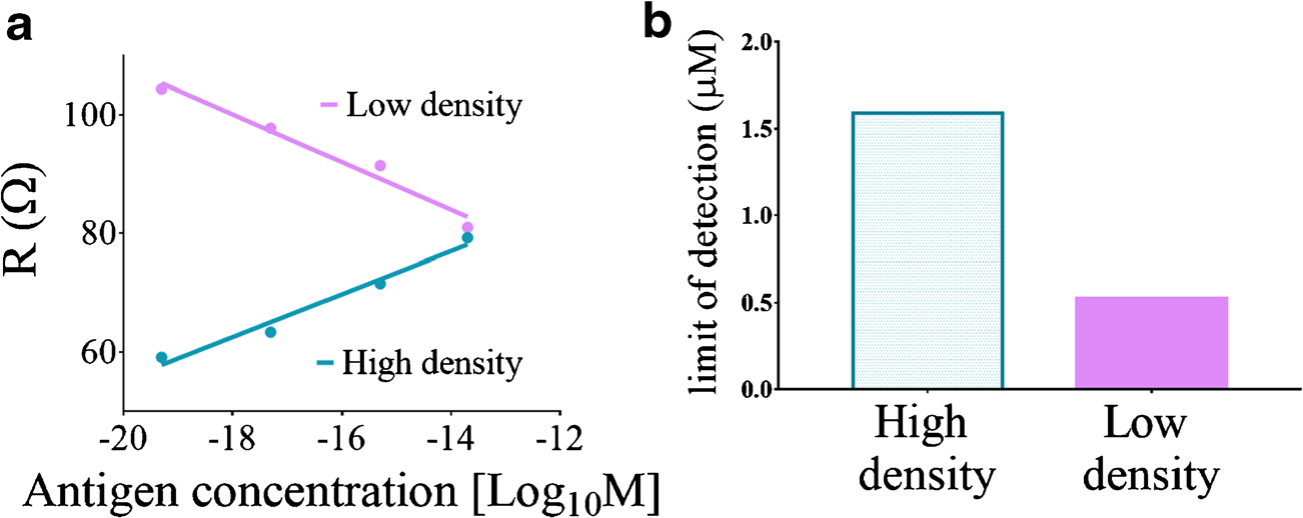
The effect of the antibody concentration of the antigen sensing performance. (**a**) Representative analysis results of the EIS measurements of the high- and low-density antibody-modified electrodes with increasing amounts of antigen (0, 50 fg/μl, 5 pg/μl, 500 pg/μl, and 20 ng/μl). The analysis was performed using the equivalent circuit mentioned in Fig. 4b. Low density is denoted by pink and high density by turquoise. (**b**) Limit-of-detection (LOD) calculations of both the high (high-density) and the low (low-density) antibody concentrations. The LOD values are expressed as three times the standard deviation of the low concentration divided by the slope of the calibration line

Next, we tried to better understand how the antibody concentration affects the bioelectronic interface and the antigen sensing performance. To this end, we used two different antibody concentrations, high (see Fig. S3A in the ESM for the recorded EIS) and low (see Fig. S3B in the ESM for the recorded EIS), and recorded EIS measurements. We analyzed the measurements with the same diffusion-restricted-based EEC model (Fig. 4b) and observed that for the high-density antibody-modified electrode, the solution resistance element increased as antigen accumulated, whereas in the low-density antibody-modified electrode, it decreased (slope: 3.6 ± 0.4 [Ω/log[M]] and Y-intercept 128 ± 7 [Ω], *R*^2^ = 0.97; slope: -4 ± 1 [Ω/log[M]] and Y-intercept: 28 ± 8 [Ω/log[M]], *R*^2^ = 0.97, respectively) (Fig. 6a). However, this kind of difference was not observed for either the constant phase element (*Q*) or the diffusion element (*R*_*ω*_) (see Fig. S3C in the ESM). We also calculated the limit of detection (LOD) for each antibody density (Fig. 6b) according to Eq. 2:

2 $$ Limit\ of\ detection=3\ast \frac{\sigma }{S}, $$

where σ is the standard deviation of the blank and *S* is the slope of the calibration curve. The LOD values were found to be 2.2 μM and 0.26 μM for the high- and low-density antibody-modified electrodes, respectively. These results show that the antibody concentration affects the detection trend measured using EIS. The positive relationship observed for the high-density antibody-modified electrode may be due to the increased antigen-antibody binding events that decreased the mass transfer flux of the electrolyte toward the electrode. Even though additional studies are required to draw conclusions, we suspect that the negative relationship observed for the low-density antibody-modified electrode may be due to the conformation change in the collapsed antibody regarding a vertical conformation upon the antigen-antibody binding event. The conformation change increased the electrode’s surface area, which is not modified with antibodies. The increased unmodified area consequently increased the mass transfer of the electrolyte unaffected by the antibodies.

## Conclusions

In this work, we examined how the antigen analyte incubation time, together with the antibody probe density at the electrode, can affect the measurement of the bioelectronic interface in a drop of solution, using a non-faradaic, diffusion-restricted, electrochemical impedance spectroscopy method. We first explored the effect of the antigen incubation time. Our results show that as the incubation time increases, our measurable values decrease. Since there are several examples in the literature in which the incubation time of the primary antibody exceeds 1 h, for this reciprocal case, we expect to observe hybridization principles that are similar to the antibody probe case studied here. Antibody probe-antigen analyte interactions reach an equilibrium state within the first 30 min [24]. Incubation times longer than 1 h ensure an equilibrium state and create a reproducible environment [25–28]. In the reciprocal case, longer incubation times can be due to the slower diffusion rate of the larger antibody towards the probe antigen. We hypothesize that the slower incubation rate can affect the response time of the immunosensor. Furthermore, since the antigen probe is smaller than the antibody, and if the antigen size is smaller than the Debye length, a stronger electrochemical signal will be measured in hybridization events, which will improve the overall sensitivity.

We tested whether and how the antibody concentration affected the bioelectronic interface. We showed that the low-density antibody-modified electrode led to a decrease in the solution resistance, whereas the high-density antibody-modified electrode led to an increase. We explained this phenomenon by examining the surface structure for each condition. We detected a thicker and rougher electrode surface as the antibody density increased. We explained these results by suggesting that the conformation of the antibody prior to the antigen-binding events is dependent on the surface density. For high antibody density, the antibodies rearrange in a vertical conformation, whereas for low antibody density the antibodies are in a horizontal conformation. Upon binding events with the antigens, the antibodies in a horizontal conformation change to a vertical conformation, increase the unmodified area of the electrode, and decrease the overall solution resistance. Our results emphasize the important effect of the incubation time and the antibody density on antibody-antigen binding events. We showed that by changing one parameter of the bioelectronic interface the sensing results could drastically change, a change that we should be aware of and that can be manipulated to achieve different detection mechanisms.

## Electronic supplementary material

ESM 1 (PDF 303 kb)
