# Supplementary material for: Probing antibody surface density and analyte antigen incubation time as dominant parameters influencing the antibody-antigen recognition events of a non-faradaic and diffusion-restricted electrochemical immunosensor
Source: Anal Bioanal Chem. 2020 Jan 29;412(7):1709–17. doi: 10.1007/s00216-020-02417-x (PMC7026205; doi:10.1007/s00216-020-02417-x)
Supplement: Supplementary file 1 — (PDF 303 kb) [file 216_2020_2417_MOESM1_ESM.pdf]

Analytical and Bioanalytical Chemistry

Electronic Supplementary Material

Probing antibody surface density and analyte antigen incubation time as dominant parameters influencing the antibody-antigen recognition events of a non-faradaic and diffusion-restricted electrochemical immunosensor

Jonathan Zorea, Rajendra P. Shukla, Moshe Elkabets, Hadar Ben-Yoav

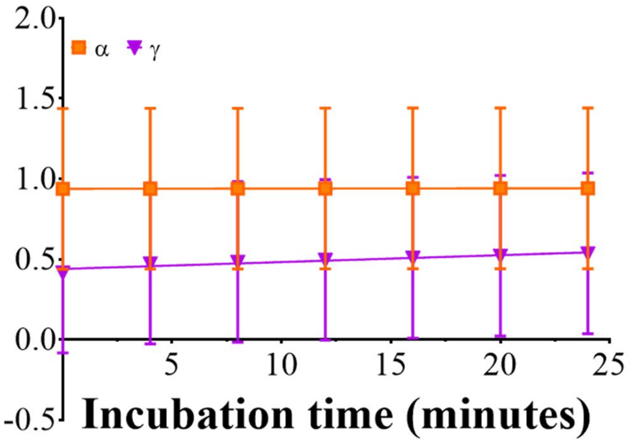

Fig. S1 The dependence of α and γ on the incubation time. Both α (orange squares) and γ (purple triangles) showed no significant dependence on the incubation duration with a very high standard deviation

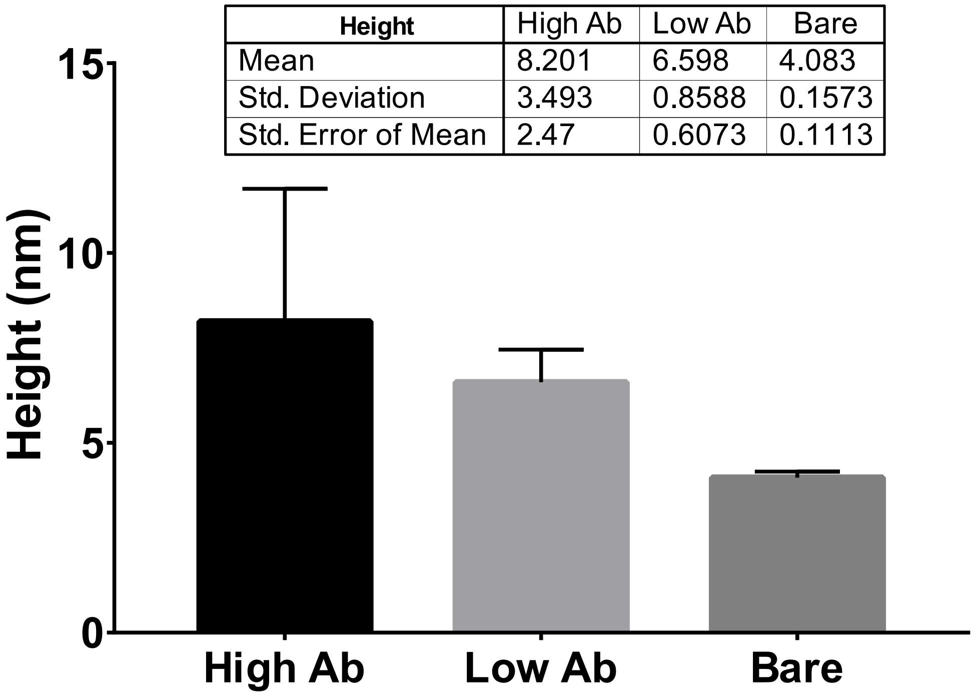

Height	High Ab	Low Ab	Bare
Mean	8.201	6.598	4.083
Std. Deviation	3.493	0.8588	0.1573
Std. Error of Mean	2.47	0.6073	0.1113

Fig. S2 Antibody height on the electrode affected by it concentration. Bar graph of the averaged height differentiating between each modification. Three separate locations on the electrode for each condition were used for the analysis process and represented as mean with SD

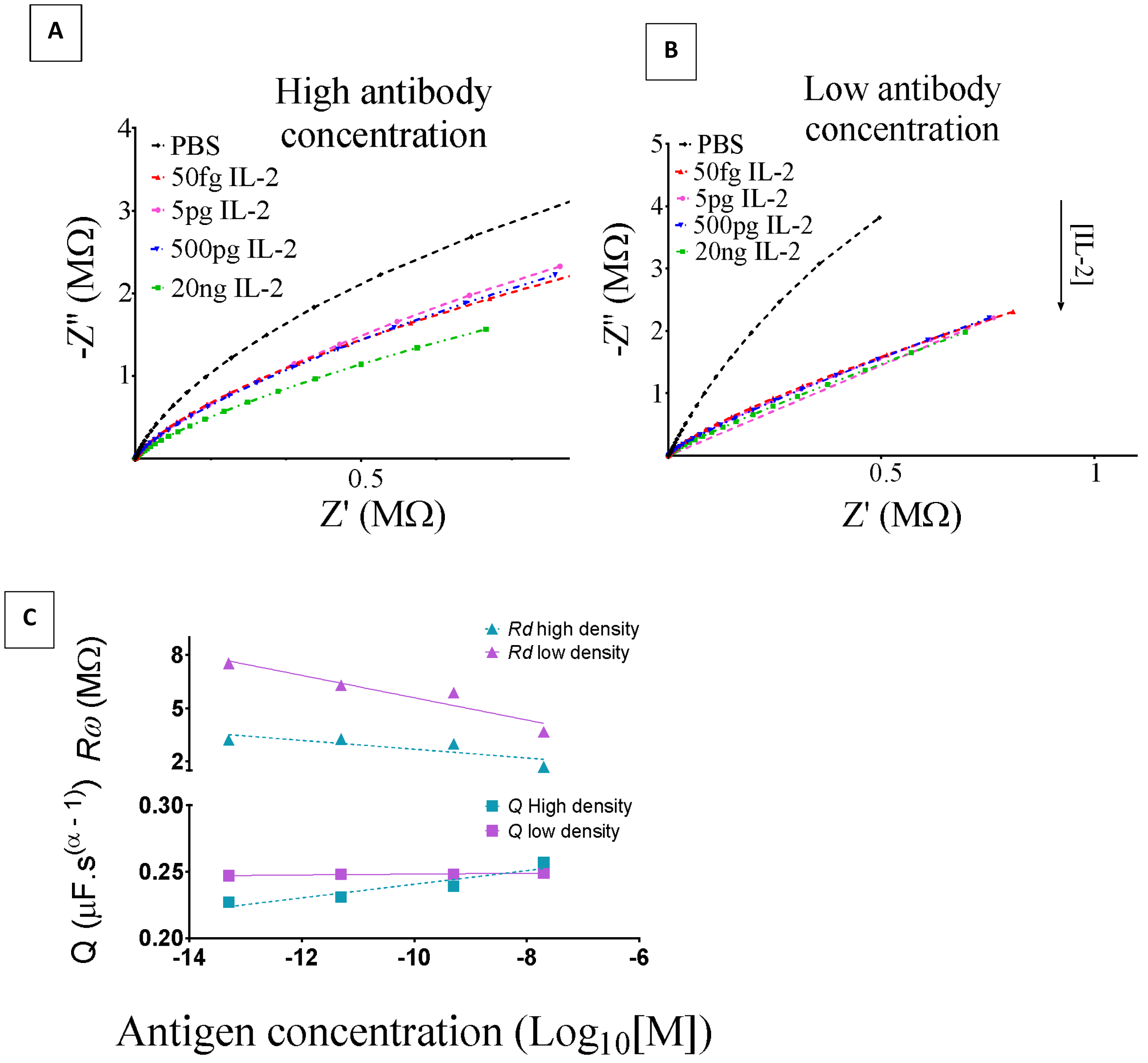

Fig. S3 The effect of the antibody concentration on the impedance measurements. Electrochemical impedance spectroscopy measurements were carried out with a fixed potential of 0.23V vs the Ag/AgCl reference electrode, at a frequency range of 1 MHz to 1 Hz and with a 25 mV amplitude and 3 measurement points per decade. (A) High antibody concentrations led to decreased impedance at low frequencies for increased antigen concentrations. (B) Low antibody concentrations led to decreased impedance at low frequencies for increased antigen concentrations. (C) Analysis of the EIS measurements of the high and low density antibody–modified electrodes with increasing amounts of antigen reveals the same slope trends in between the high (dashed line) and low (solid line) density electrodes for both the constant phase element apacitive magnitude (*Y*; squares) and the diffusion element (R_{ω}; triangles)
